# Gene regulatory network analysis identifies MYL1, MDH2, GLS, and TRIM28 as the principal proteins in the response of mesenchymal stem cells to Mg^2+^ ions

**DOI:** 10.1016/j.csbj.2024.04.033

**Published:** 2024-04-14

**Authors:** Jalil Nourisa, Antoine Passemiers, Farhad Shakeri, Maryam Omidi, Heike Helmholz, Daniele Raimondi, Yves Moreau, Sven Tomforde, Hartmuth Schlüter, Bérengère Luthringer-Feyerabend, Christian J. Cyron, Roland C. Aydin, Regine Willumeit-Römer, Berit Zeller-Plumhoff

**Affiliations:** a Institute of Material Systems Modeling, Helmholtz Zentrum Hereon, Geesthacht, Germany; b ESAT-STADIUS, KU Leuven, Heverlee, Belgium; c Institute of Medical Biometry, Informatics and Epidemiology, Medical Faculty, University of Bonn, Bonn, Germany; d Institute of Clinical Chemistry/Central Laboratories, University Medical Center Hamburg, Hamburg, Germany; e Institute of Metallic Biomaterials, Helmholtz Zentrum Hereon, Geesthacht, Germany; f Department of Computer Science, Intelligent Systems, University of Kiel, Kiel, Germany; g Institute of Clinical Chemistry and Laboratory Medicine Diagnostic Center, University of Hamburg, Hamburg, Germany; h Institute for Continuum and Material Mechanics, Hamburg University of Technology, Hamburg, Germany

**Keywords:** Gene regulatory network analysis, Mesenchymal stem cells, Magnesium ions, Proteomics

## Abstract

Magnesium (Mg)-based implants have emerged as a promising alternative for orthopedic applications, owing to their bioactive properties and biodegradability. As the implants degrade, Mg^2+^ ions are released, influencing all surrounding cell types, especially mesenchymal stem cells (MSCs). MSCs are vital for bone tissue regeneration, therefore, it is essential to understand their molecular response to Mg^2+^ ions in order to maximize the potential of Mg-based biomaterials. In this study, we conducted a gene regulatory network (GRN) analysis to examine the molecular responses of MSCs to Mg^2+^ ions. We used time-series proteomics data collected at 11 time points across a 21-day period for the GRN construction. We studied the impact of Mg^2+^ ions on the resulting networks and identified the key proteins and protein interactions affected by the application of Mg^2+^ ions. Our analysis highlights MYL1, MDH2, GLS, and TRIM28 as the primary targets of Mg^2+^ ions in the response of MSCs during 1–21 days phase. Our results also identify MDH2-MYL1, MDH2-RPS26, TRIM28-AK1, TRIM28-SOD2, and GLS-AK1 as the critical protein relationships affected by Mg^2+^ ions. By offering a comprehensive understanding of the regulatory role of Mg^2+^ ions on MSCs, our study contributes valuable insights into the molecular response of MSCs to Mg-based materials, thereby facilitating the development of innovative therapeutic strategies for orthopedic applications.

## Graphical abstract

**Figure:**
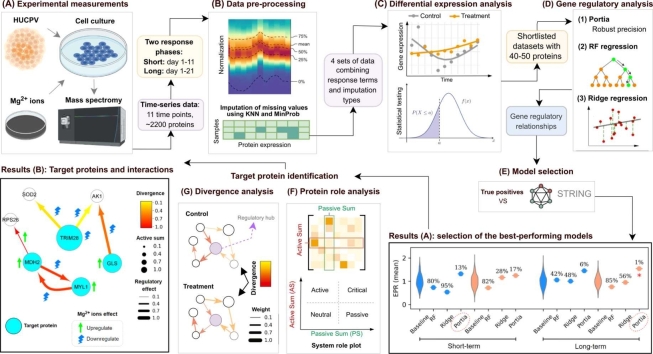

## 1 Introduction

Magnesium (Mg)-based alloys are becoming increasingly popular in the orthopedic industry due to their biodegradable and bioactive properties [1]. After insertion into the body, implants made from Mg alloys degrade and release certain products, such as Mg^2+^ ions. These interact with the local environment and regulate various physiological processes, particularly the activities of Mesenchymal stem cells (MSCs) [1], [2]. MSCs play a crucial role in bone tissue regeneration. They migrate to sites of bone damage, proliferate to increase cell population, and differentiate into various cell types essential for bone repair and remodeling, such as osteoblasts [3]. MSCs also modulate the inflammatory response by interacting with immunocompetent cells throughout the bone regeneration process [4], [5], [6]. MSCs have been the focus of numerous studies examining their response to the degradation products of Mg biomaterials [2], [7], [8], [9]. A growing body of empirical evidence highlights the essential role of Mg^2+^ ions in stimulating diverse cellular activities in MSCs, such as proliferation and osteogenic differentiation [7], [10], [11]. Despite these findings, our comprehension of the mechanisms governing the regulatory impact of Mg degradation products on MSCs is still incomplete. To fully harness the potential of Mg-based biomaterials for orthopedic applications, it is pivotal to acquire a thorough understanding of how these materials influence intracellular processes.

Proteomics is a powerful method used to identify, quantify, and analyze proteins and their interactions within a given system or in response to certain stimuli [12], [13]. This method enables the generation of extensive datasets containing expression profiles of hundreds to thousands of proteins [13]. Different statistical methods and machine learning approaches are used to unravel the complexity of the proteomics data such as differential expression (DE) analysis and gene regulatory network (GRN) analysis [14], [15], [16]. Statistical tests illuminate proteins with significant changes in expression levels, indicative of e.g. a treatment response. For time-series data, the progression of expression over time can be taken into account using time-series DE analysis, thereby capturing the dynamic shifts in protein expression patterns [17]. GRN analysis seeks to study inter-gene interactions and to gain mechanistic insights into the causal relationships between genes [18]. A variety of methods have been proposed for this purpose such as Boolean networks, information-theoretic approaches, and feature selection methods [18], [19], [20]. Regression analysis is a popular example of feature selection methods, successfully employed using linear models such as Lasso [21] and Ridge [22] models and non-linear models such as Random Forests (RF) [23]. More recently, Passemiers et al. proposed Portia [24], a GRN inference method using robust precision matrix estimation. They showed that this method outperforms many well-known benchmarking techniques in reconstructing a GRN [24].

Multiple proteomics studies have been undertaken to examine the bioregulatory effect of Mg-based materials on the molecular response of MSCs [16], [25], [26]. For instance, Sánchez et al. [25] conducted a proteomics analysis to investigate the impact of Mg-based materials degradation on MSCs undergoing chondrogenesis. They characterized DE proteins and biological functions associated with Mg^2+^ ions using enrichment analysis [25]. Omidi et al. [26] examined the proteomic response of MSCs to Mg^2+^ ions and revealed their regulatory effect on the proteins involved in extracellular matrix maturation and remodeling [26]. While these studies provide information about the effects of Mg^2+^ ions, they did not explore the intricate protein regulatory networks modulated by Mg^2+^ ions. Moreno et al. employed GRN analysis to study the effects of Mg^2+^ ions on the regulatory network within initial 11 days after exposure to Mg^2+^[16]. They used a linear GRN method to model protein-protein regulatory interactions and conducted Vester’s sensitivity analysis [27] to assess the influence of Mg^2+^ ions on protein regulatory roles within the network [16]. They pinpointed two proteins of S100A10 and PABPC4 as the key targets of Mg^2+^ ions during the first 11 days of MSCs response [16].

In this study, our objective is to investigate the pivotal proteins and their interactions that critically modulate the response of MSCs to Mg^2+^ ions using GRN analysis. We consider two response phases for MSCs: short-term (the initial 11 days) and long-term (up to 21 days). These intervals are pivotal for the involvement of MSCs in bone repair; within the first two weeks, MSCs migrate to the injury site, modulate inflammation, and initiate chondrogenic differentiation [3]. Subsequently, in the healing continuum, MSCs progress to osteogenic differentiation and commence the calcification of the soft callus [3]. For the short-term response, we analyze sequential proteomic data across 9 time points from day 1 to day 11, similar to Moreno et al. For the long-term response, we extend our observation to include 11 time points, incorporating additional data from days 14 and 21. We employ various statistical models and GRN methods, encompassing regression analysis and a precision matrix approach, to construct intricate molecular representations of MSC responses to Mg^2+^ ions. By evaluating these resultant networks, we examine the complex molecular interplay and regulatory mechanisms underpinning the influence of Mg^2+^ ions on MSC behavior.

## 2 Methods

**Fig. 1:**
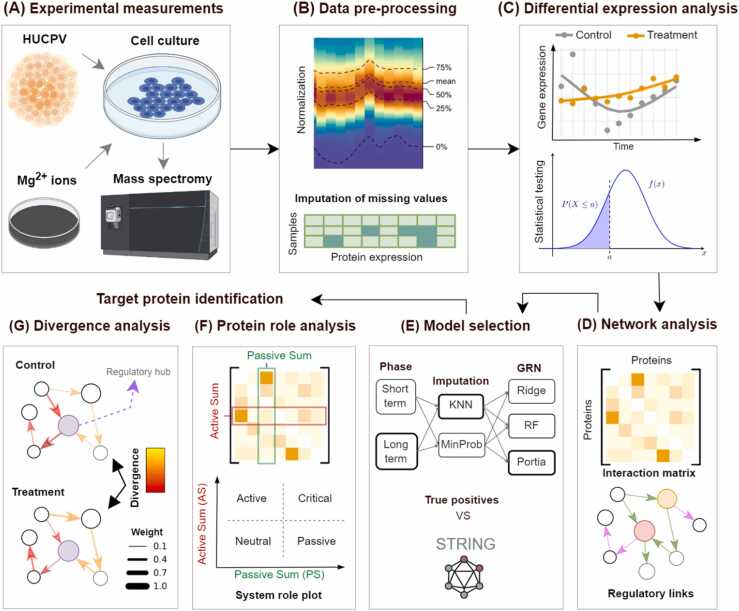
The overview of the methodology implemented in this study. (A) Proteomics data were collected for the control and treatment groups, where the treatment group was exposed to 5 mM of Mg^2+^ ions. (B) The proteomics data were normalized to remove potential biases, and missing values were imputed using two the methods of KNN and MinProb. (C) Time-series analysis was performed to identify the most sensitive proteins to Mg^2+^ ions. (D) Network analysis was performed to reveal protein regulatory connections. (E) Model selection was employed to choose the best-performing GRN model according to the known links provided by the STRING database. (F) Protein role analysis was employed to obtain the proteins’ regulatory roles and to determine the proteins with a significant role change in response to Mg^2+^ ions. (G) Protein regulatory divergence analysis was performed to detect the regulatory links most affected by Mg^2+^ ions. Target proteins were identified using the cumulative information of F and G.

Fig. 1-A gives a schematic overview of the employed methods and workflow in this study. In the cell culture experiments, Human Umbilical Cord Perivascular (HUCPV) cells were supplemented with or without 5 mM of Mg extracts, designated as treatment and control groups, respectively, and the time-series proteomics data was acquired using mass spectrometry at 11 time points presenting measurements on 2229 proteins (see section ‎2.1). We divided the data into two phases: short-term (days 1–11) and long-term (days 1–21), with 9 and 11 sample points for the former and latter, respectively. The data was subsequently normalized, and the missing values were imputed using K-Nearest Neighbors (KNN) and Probabilistic Minimum Imputation (MinProb) techniques (see Fig. 1-B and section ‎2.2).

We carried out a time-series DE analysis to pinpoint proteins that displayed distinct changes in response to Mg^2+^ ions (see Fig. 1-C and section ‎2.2). This approach enabled us to identify the proteins for further investigation, reducing the complexity of our ensuing analysis. Subsequently, we evaluated the impact of Mg^2+^ ions on the interplay between these DE proteins using GRN analysis (see Fig. 1-D). We chose three different GRN inference methodologies: Ridge (linear regression utilizing the Ridge model), RF (non-linear regression executed with random forest regression), and Portia (a robust precision matrix estimation procedure). Refer to sections ‎2.3 and ‎2.4 for further details about these methods. Overall, our approach led to the creation of 12 distinct models, factoring in the four datasets and the three GRN inference strategies (see Fig. 1-E).

After, we conducted model selection on the created GRN models and chose the two best-performing models, one for each response phase of short-term and long-term for further analysis (refer to section ‎2.5). Then, we utilized two distinct approaches to extract information from the inferred links in the network. Firstly, we conducted protein role analysis (PRA) to ascertain the regulatory importance of proteins and the impact of the Mg^2+^ ions treatment on them (see Fig. 1-F and section ‎2.7). Through this analysis, we narrowed down the proteins that exhibited significant role changes in response to Mg^2+^ ions. Secondly, we analyzed the protein regulatory relationships and the influence of Mg^2+^ ions on them using protein regulatory divergence analysis (PRDA) (see Fig. 1-G and section ‎2.8). Through this analysis, we identified the most critical regulatory links affected by Mg^2+^ ions, and subsequently determined the proteins with the central role in the network. Finally, we identified the target proteins by considering both evaluation perspectives and incorporating cross-validation.Top of Form The code and the data of this study is available online [28].

### 2.1 Sample preparation and proteome extraction

The proteomic time-series data utilized in our analysis encompasses measurements taken at 11 distinct time points: days 1, 2, 3, 4, 7, 8, 9, 10, 11, 14, and 21. The dataset from days 1 to 11 was initially published by Moreno et al. [16], and our analysis expanded this to also include measurements on days 14 and 21. This extension allowed us to study the long-term response of MSCs to Mg^2+^ ions across a 21-day period. It should be noted that these measurements are all from the same experiment, and therefore there were no batch effects. HUCPV cells, originated from the Wharton's jelly surrounding the vessels in the umbilical cord, were selected for cell culture experiments. HUCPV cells exhibit comparable differentiation capabilities to MSCs derived from bone marrow, with the added advantage of relatively higher proliferation rates in lower passaging numbers [29], [30].

Cells at passage 3 were cultured for up to 21 days either in cell culture media or media supplemented with the Mg extract. Dulbecco's Modified Eagle Medium (DMEM) containing 10% fetal calf serum (FCS) was utilized as the culture medium. At different time points the cells were washed three times with phosphate-buffered saline (PBS), trypsinized, washed three times again with PBS, snap-frozen, and stored at − 80 °C. To extract the proteins, the cell pellets were denatured, digested in a tryptic solution, and desalted. LC-MS/MS was performed using a nano-flow UPLC (DionexUltiMate 3000 RSLCnano, Thermo Fisher Scientific, Bremen, Germany) and an Orbitrap mass spectrometer (Orbitrap-Fusion, Thermo Fisher Scientific), which were coupled via electrospray ionization (ESI). The SwissProt database which was downloaded from UniProt in July 2015 and an internal contaminants database were used for identification and label-free quantification, with MaxQuant 1.5.2.8 processing the mass spectrometric data. The identification parameters included a precursor mass tolerance of 20 ppm, fragment mass tolerance of 0.5 Da, PSM and protein FDR of 0.01, and a minimum peptide length of 6 amino acids for identification using a match between runs. At least two ratio counts were considered for protein quantification, with specific digestion mode and trypsin as an enzyme with one missed cleavage allowed for peptide identification. The complete description of the proteome extraction is provided by Moreno et al. [16].

### 2.2 Data preprocessing and time-series differential expression analysis

To ensure reliable downstream analysis, the data underwent two the preprocessing steps of (1) base-2 log transformation and (2) global median normalization. The log transformation helped to reduce the impact of extreme values. In addition, a normalization step was implemented to equilibrate the median across all samples, thereby mitigating the impact of potential biases. This procedure underscores our primary focus on the relative disparities among the groups as opposed to their absolute values. This preprocessing is essential for DE analysis and regression modeling, which rely on unbiased and normalized data. Next, we excluded those proteins with more than 50% missing values across samples. To handle missing values in the remaining proteins, we employed two imputation methods: KNN and MinProb. The KNN method assumes that the missing values are randomly distributed in the data, while the MinProb method assumes that the missing values are due to low protein abundance in certain samples [31]. Since missingness in reality can result from a combination of both assumptions, we implemented both methods and used model selection to determine the best-performing method. We used the R package imputeLCMD with default parameter values for the imputation [32]. To clearly label the generated datasets, we used a naming convention encompassing the response phase and imputation method, and GRN method. For example, a dataset would be denoted as LongTerm-KNN.

In the subsequent phase, we performed a DE analysis on the time-series data to pinpoint the proteins with distinct responses to Mg^2+^ ions. To this end, we fitted spline curves to the expression data of each protein within individual groups of control and treatment (see Fig. 1-C). We utilized natural cubic regression splines with three degrees of freedom, using the splines library in R [33]. Next, we implemented the empirical Bayes-moderated F-statistic [34] to evaluate the differential expression between the two groups, based on the estimated parameters of the fitted models. Finally, we corrected the resulting p-values for multiple testing using the Benjamini-Hochberg procedure, which enabled us to compute the false discovery rates [35]. This process was carried out using the 'limma' R package [36]. Significance threshold was set at 0.05.

### 2.3 GRN analysis using regression models

For regression models, we modified the design of dynGENIE3 which allows for hyper-parameter optimization. This Python framework also supports regression analysis using other models such as Ridge and Lasso. The source code is accessible in Zenodo repository [37].

#### 2.3.1 Time-discrete formulation

**Fig. 2:**
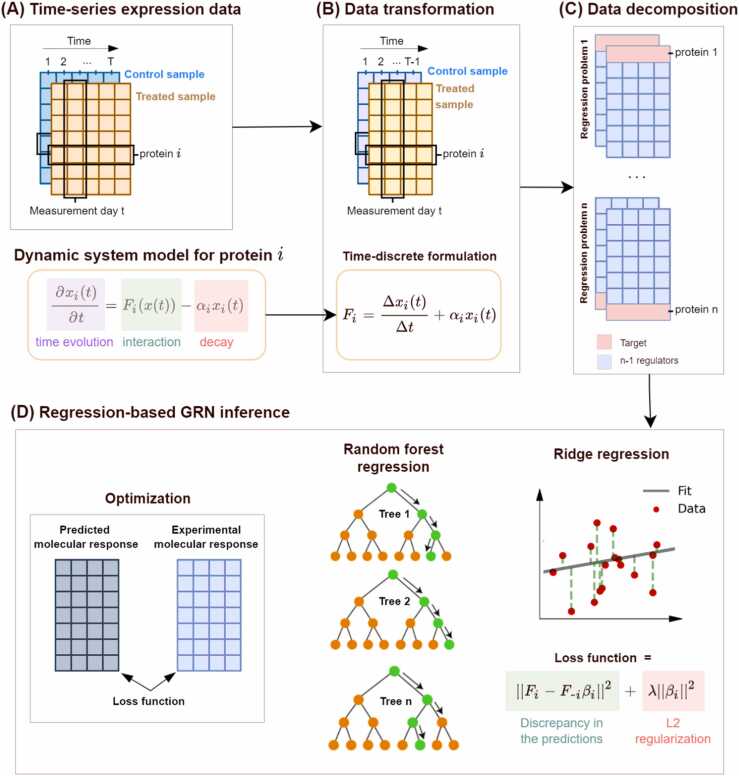
The protocol of GRN inference using regression analysis. Time-series data (A) was discretized to obtain learning samples of $F_{i}$ (B). Next, the data was partitioned into sets of features and targets for each protein (C). Then, *n* regression models were trained using the expression data of *n* proteins in the network using RF and Ridge models (D).

In the regression analysis, we simulated the time-dependent expression of proteins using ordinary differential equations (Fig. 2-A), similar to dynGENIE3 [38] and CellBox [39]. Then, we transformed the time-continuous formulation into a time-discrete one to enable regression analysis [38], [39] (Fig. 2-B). We modeled the dynamic evolution of protein expression using Eq. (1), where $x_{i}$ is the expression level of protein *i*, $\alpha_{i}$ is the protein’s decay rate, and $F_{i}$ is the non-linear function predicting the expression level of protein *i* based on the concentration of all of the proteins in the network (*x*) [38].

(1) $$\frac{\partial x_{i}(t)}{\partial t}=F_{i}\left(x\left(t\right)\right)-\alpha_{i}x_{i}(t)$$

By discretization in time (with *T* the number of measurement points in time) and finite difference approximation of Eq. 1, we obtain:

(2) $$F_{i}\left(x\left(t_{k}\right)\right)=\frac{x_{i}\left(t_{k+1}\right)-x_{i}\left(t_{k}\right)}{t_{k+1}-t_{k}}+\alpha_{i}x_{i}\left(t_{k}\right),k=1\mathrm{to}T-1$$

By discretizing Eq. (1) as Eq. (2), the time-series data is converted into learning samples (see Fig. 2-B), where regression models can be directly applied to infer $F_{i}$. In Eq. (2), the decay coefficients $\alpha_{i}$ can be either obtained from the literature, if available, or estimated as model hyperparameters. We infer these hyperparameters from training data during hyperparameter tuning.

#### 2.3.2 Regression models for GRN inference

Next, we partitioned the expression data into sets of features and targets for each protein; for protein *i*, we used its expression data as the target and the expression data of the rest of the proteins as the features (regulators) [23], [38], [40] (Fig. 2-C). We trained *n* regression models independently using the expression data of *n* proteins in the network (Fig. 2-D). For this purpose, we utilized the two regression models of RF and Ridge. Ridge and RF are both established regression models in the literature for inferring GRN through regression analysis [23], [38], [40], [41]. In Ridge models, the targets are modeled as a regularized linear combination of the input features, while RF provides non-linear modeling using randomized decision trees. RF and Ridge are both capable of handling high-dimensional datasets, where the number of features exceeds the sample size, thanks to their regularization mechanisms.

#### 2.3.3 Hyperparameter calibration

To improve the prediction accuracy and extract meaningful regulatory links, we first calibrated the model hyperparameters, including the decay coefficients of proteins and the hyperparameters of the regression estimators. As for the latter, we tuned *alpha* for the Ridge models and *max _features* for the RF models. We used the out-of-bag (OOB) score and leave-one-out (LOO) score for the cross-validation of RF and Ridge models, respectively. The OOB score is the prediction score on the samples that were not included in the construction of a given tree. Due to the stochasticity of the RF model, we repeated the tuning process ten times and took the average values of the resulting scores. In the LOO approach, we trained the model on all but one of the samples in the dataset and used the remaining sample for testing. This process was repeated for each sample in the dataset, and the resulting performance scores were averaged.

We used the coefficient of determination (R^2^) as the evaluation measure to assess the performance of the regression models. R^2^ represents the proportion of variance in the target variable that can be explained by the independent variables (features or regulators). It is calculated according to Eq.(3), where SSR is the sum of squared residuals (differences between the predicted values and the actual values) and SST is the total sum of squares (squared differences between the actual values and their mean).

(3) $$R^{2}=1-\left(\frac{\mathrm{SSR}}{\mathrm{SST}}\right)$$

#### 2.3.4 Inference of regulatory importance

In GRN inference using regression analysis, feature importance serves the purpose of determining the importance of regulator genes or proteins in the network [23], [40], [42]. In Ridge regression, the absolute values of the coefficients of the linear regression model ($\beta_{j}$ in Fig. 2-D) indicate that feature’s importance. The higher the absolute value of a coefficient, the more important the corresponding feature. In RF regression, the importance of a feature is calculated by measuring the mean decrease in impurity, which represents the average reduction in the impurity of the target variable across all decision tree nodes where the feature is used to split the data. To enhance the comparability and interpretability of feature importance scores across multiple regression models, we normalize the scores by dividing the score of each feature by the sum of scores for all features in the same model [23], [43]. This results in the relative importance of each feature within the given model.

### 2.4 GRN analysis using precision matrix estimation

Covariance matrices are often used to capture correlations between genes in a network, where each element represents the covariance between genes *i* and *j* [44]. However, using the content of covariance matrices to directly infer regulatory links can lead to a high proportion of false positives [24]. To overcome this limitation, precision matrices, which are the inverse of covariance matrices, can be used instead [44]. Precision matrices have a strong mathematical basis and are statistically interpretable as they define a multivariate Gaussian distribution over the expression data. Importantly, the element *(i, j)* of a precision matrix is equal to 0 when genes *i* and *j* are conditionally independent given the rest of the network, indicating that no direct regulatory association exists between the two genes after accounting for indirect effects [45]. Therefore, by using precision matrices, direct correlations between genes can be disentangled from indirect correlations, leading to identifying regulatory links with higher accuracy [44]. This approach allows us to distinguish between direct regulatory associations and indirect effects that result from multiple intermediate genes.

In our study, we utilized the robust precision matrix estimation method implemented in Portia [24] to accurately reconstruct GRNs. To verify the assumption of a Gaussian distribution underlying the precision matrix approach, we first power-transformed the data, similar to the implementation of Portia. Then, we conducted a normality test using the *normaltest* function from Scipy [46]. Our analysis revealed a p-value of 0.62, confirming that the data follows a normal distribution. Portia performs post-processing steps on the precision matrix to ensure its relevance for accurate GRN reconstruction. Specifically, it uses a shrinkage estimation of the covariance matrix to address numerical issues arising from the under-determination of the problem when the number of samples is smaller than the number of genes [47]. We adopted the default value of the parameter *λ,* which determines the regularization strength in Portia [24]. In addition to regularization, Portia applies correction steps to alleviate the symmetry of precision matrices, and correct for expression biases by row- and column-wise correction [24]. As Portia does not explicitly model time series, we directly used the expression samples without converting them to the time-discrete format (see ‎2.3).

### 2.5 Model selection

We labeled the generated models using a naming convention encompassing the response phase, imputation method, and GRN method. For example, a model would be denoted as LongTerm-KNN-Portia. We adopted a model selection process to choose the models with adequate information about the regulatory system using two key metrics: the prediction score denoted by the R^2^ score (only for regression models) and the Early Precision Ratio (EPR). The R^2^ score indicates the model's capacity to capture the information inherent in the data, while the EPR serves as an indicator of how accurately the deduced networks mirror established protein functional relationships. For regression-based models, we began by evaluating their predictive performance using the R^2^ score before advancing to GRN inference. It is crucial that a regression model effectively learns and predicts expression data before analyzing the resulting feature importance.

Subsequently, we evaluated the performance of the models in retrieving the known links using the EPR. We obtained protein functional relationships from the STRING database (version 11.5), which is a comprehensive resource on protein networks, and used them as ground truth to evaluate the inferred models [20], [48]. Since the inferred links are continuous values, to determine the true positives, we created a binary network from the inferred interaction weights by selecting the most important interactions suggested by the model, referred to as putative regulatory links [23], [49]. For a given protein-protein interaction, this process results in either a 1, indicating the presence of a regulatory relationship, or a 0 for no interaction. However, instead of relying on a single selection of putative links, similar to [39], we conducted this comparison for ten different selections from the top 75th to 90th percentile of the inferred links, and averaged the resulting scores. This approach is more robust compared to a single threshold comparison, as it reduces the likelihood of obtaining higher scores due to artifacts in the choice of putative links. We then computed the Early Precision (EP) score, which represents the percentage of known links accurately captured by the model. Next, we calculated EP scores for 1000 randomly generated models to serve as baseline scores. The relative success of a GRN inference method was determined using the EPR, which is the corresponding EP score normalized to the baseline score.

### 2.6 Robustness analysis

We conducted a robustness analysis to evaluate the influence of small variations in the protein expression data on the results. To model experimental uncertainty, we applied different types of noise to the protein expression data and evaluated the effect of the introduced noise on the expected results. Multiplicative Gaussian noise (MGN) and additive Gaussian noise (AGN) were used to generate noise as shown in (4), (5), respectively, where *x* is the experimental measurement, N(1,σ) is the Gaussian distribution with a mean of 1 and a standard deviation of σ, and $x^{*}$ is the generated noisy data [39].

(4) $$x^{*}=x+N(0,\sigma_{a})$$

(5) $$x^{*}=x\times N(1,\sigma_{m})$$

In this study, we utilized standard deviation values of 0.05 and 0.1 for $\sigma_{m}$ and $\sigma_{a}$ corresponding to MGN and AGN at 5% and 10% of the variance of the actual data, respectively. These values were derived based on the findings of Yuan et al., who observed a significant decline in GRN inference accuracy beyond 5% MGN and 10% AGN levels [39].

### 2.7 Protein role analysis

In order to gain a deeper understanding of the function of proteins in the regulatory network, we employ PRA using Vester’s sensitivity analysis (VSA) [27]. VSA receives an impact matrix that contains the regulatory interactions between proteins and characterizes each protein’s role (see Fig. 1-F). To eliminate low-impact and potentially noisy connections, we selectively incorporate the top 75% of the inferred links into the impact matrix. Then, we calculate two primary parameters of active sum (AS) and passive sum (PS), which represent the regulatory importance of protein (element) on the rest of the network and how a protein is impacted by the rest of the network, respectively (see Fig. 1-F). Visualizing AS versus PS in the system role plot helps characterizing proteins based on their participation in the regulatory network. Highly active variables are situated in the upper-left corner of the plot, while highly reactive variables are located in the lower-right corner. Similarly, highly critical and neutral variables are located in the upper-right and lower-left corners, respectively.

We use the (AS, PS) coordinates to calculate the changes in protein roles between control and treatment groups. We characterize two types of significant role changes: (1) those that experience a large change in their roles between the two groups, and (2) those that experience a role change from critical to another role. To define the critical role, we mark the AS and PS axes by the 75th percentiles, and identify those that simultaneously exceed these thresholds on both the AS and the PS axes. In the following, we determine the significant role changes across the groups. To this end, we conduct a robustness analysis according to section ‎2.6 to obtain the distribution of roles for each protein within two groups. Then, we conduct a t-test with a threshold of 5% to identify the proteins with significantly different roles across the groups. Proteins exhibiting significant changes across both MGN and AGN conditions were deemed robust.

### 2.8 Protein regulatory divergence analysis

We conducted PRDA to identify the protein regulatory relationships critically affected by the application of Mg^2+^ ions (see Fig. 1-G). To this end, we first calculated the differences in the inferred regulatory links between the control and treatment groups, subsequently identifying the top 10% of the connections with the largest changes. Next, we conducted a robustness analysis, according to section ‎‎2.6, to create a distribution for each regulatory connection within both groups. Then, we calculated the divergence scores across the groups using the Jensen-Shannon method. This calculation rendered a quantifiable measure of alteration in the regulatory links in response to Mg^2+^ ions.

## 3 Results and discussion

In this section, we first present the results of DE analysis, highlighting that around 40 to 50 proteins showed significance across different datasets using normal p-values (section ‎3.1). Next, we construct different GRN models using these identified proteins and select two models of ShortTerm-MinProb-Portia and LongTerm-KNN-Portia, respectively for short- and long-term phases (section ‎3.2). We then demonstrate that the selection of DE proteins for our GRN models surpasses the performance of models utilizing randomly chosen proteins, providing evidence of the effectiveness of our DE analysis in identifying important proteins (section ‎3.3). Additionally, we establish that the EPR scores obtained for our selected models are comparable to those achieved in analogous studies with more extensive datasets (section ‎3.4).

Next, we identify ACO1, HDGFL2, and MYL1 as the primary protein targets of Mg^2+^ ions for short-term response, and MDH2, TRIM28, MYL1, and GLS for the long-term response, using PRA and PRDA analyses (section ‎3.5, ‎3.6, and ‎3.7). We provide background information regarding the significant target proteins and their established biological roles documented in the existing literature (section ‎3.8). We also present findings related to their direct or indirect associations with Mg^2+^ ions. Next, we show important protein-protein interactions affected by Mg^2+^ ions, which could be potentially responsible in directing Mg^2+^ ions in affecting cellular behaviors (section ‎3.9). More notably, MDH2-MYL1, MDH2-RPS26, TRIM28-AK1, TRIM28-SOD2, and GLS-AK1. Lastly, we address the limitations inherent in our dataset, along with the underlying assumptions and methodologies utilized in this study, while also proposing potential improvements in future work (section ‎3.10). Throughout this study, we refer to proteins by their gene names for improved readability.

### 3.1 Missingness, differential expressed proteins, and GRN analysis

**Fig. 3:**
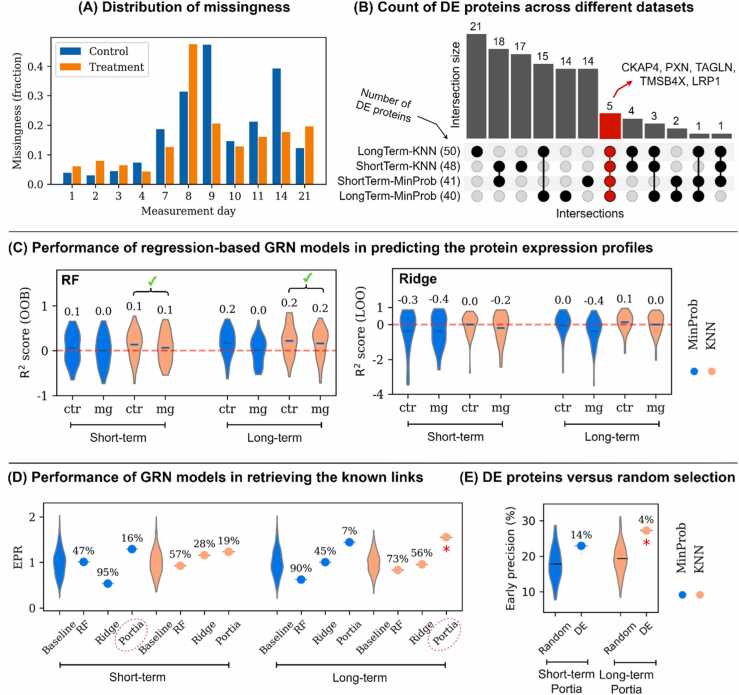
(A) The distribution of the missingness across different samples and measurement days. (B) The count of the DE proteins identified for different datasets and their interactions. Gene names are used instead of protein names. (C) Prediction scores of the protein expression for the regression-based models of RF and Ridge. Those models indicated as ✓ outperform the baseline random models (red dashed line). (D) The performance of different models in recovering the known links, measured by EPR score. The baseline models represent the scores obtained for the 1000 randomly generated networks. The numbers in percentage indicate the percentile rank of each model compared to the random models. The models chosen for short- and long-term responses are distinguished by their names encircled in a red oval. (E) The performance of the selected models of ShortTerm-MinProb-Portia and LongTerm-KNN-Portia compared to 100 models built using randomly chosen proteins as regulators. Red asterisks (*) represent the top 5% percentile ranks.

The distribution of the missingness across different samples are shown in Fig. 3-A. We observe relatively higher missingness in days 8, 9, and 14 compared to the rest of time points. We did not observe a notable difference between control and treatment. The results of DE analysis are shown in Fig. 3-B. The DE analysis results did not yield any significantly DE proteins based on the adjusted p-values. Therefore, we relied on nominal p-values to identify DE proteins. Depending on the dataset, we detected 40 to 50 DE proteins (Fig. 3-B). We identified a greater number of DE proteins in datasets created using KNN compared to those using MinProb. Only five proteins were found to be significant across all datasets, namely CKAP4, PXN, TAGLN, TMSB4X, and LRP1 (Fig. 3-B). Subsequently, we employed these DE proteins to construct GRN models. The outcomes shed light on the regulatory relationships between protein pairs within the network, leading to the generation of interaction weights that underline the relative importance of each interaction. We determined putative regulatory links by setting a cut-off threshold ranging between the 75th and 90th percentiles. These inferred links were then compared with known links during the process of model selection.

### 3.2 Model selection

Initially, we evaluated the performance of the regression-based models in predicting protein expression data. Our results revealed high variation in the prediction scores of the regression models designed for the secretion profiles of different proteins, as depicted in Fig. 3-C. Each data point in the plot represents the score in predicting the expression profile of a single protein. While some proteins were accurately predicted with a R^2^ scores of close to 1, the models failed to perform better than a random baseline model for certain proteins. Overall, RF models demonstrated superior performance in comparison to Ridge models, as evidenced by higher R^2^ scores. Furthermore, the models trained on control samples yielded better R^2^ scores than those trained on samples treated with Mg^2+^. Additionally, models utilizing the KNN imputation method outperformed those using the MinProb method. We used the R^2^ scores as the primary model selection criterion and filtered out the models that produced inferior results compared to the baseline model by taking into account both control and treatment groups.

As a result, only 2 out of 8 regression models, i.e. ShortTerm-MinProb-RF and LongTerm-KNN-RF, marginally outperformed the baseline models (see Fig. 3-C). This finding suggests that the regression models were not entirely successful in learning the complexity of protein expressions in our study. The limited performance of the regression models could be attributed to our small sample size, as these methods have been demonstrated to be successful on several benchmarking datasets with substantially larger sample sizes [23], [50]. This is further evidenced as the models based on long-term datasets, with more sample sizes, yielded superior R^2^ scores compared to the short-term counterparts (see Fig. 3-C).

Next, we calculated the EPR scores for all models as shown in Fig. 3–D. The percentile rank demonstrates the relative performance of the model among the 1000 randomly generated models. In general, models from the long-term period achieved higher EPR scores and rankings compared to their short-term counterparts. The EPR scores further implies that the regression models were not suitable for our data as they exhibited limited success in accurately inferring protein regulatory relationships. On the other hand, Portia consistently outperformed both the regression-based models and the baseline random models across all datasets. Notably, Portia produced a percentile rank of 1% for the dataset LongTerm-KNN. This finding highlights the suitability of the precision matrix-based method of Portia in uncovering protein relationships for our study with a low sample size.

By taking into account the EPR scores, we selected two top-performing models, one for each response phase. This resulted in ShortTerm-MinProb-Portia and LongTerm-KNN-Portia. To discern their superiority over baseline models, we established a 5% threshold, which could be indicative of significant model performance. This criterion suggests that our selected model encapsulates pertinent information regarding the regulatory system while maintaining a low probability of producing false results. Upon analysis, only LongTerm-KNN-Portia exhibited significant superiority, as shown in Fig. 3–D. Conversely, ShortTerm-MinProb-Portia only marginally outperformed random models with a percentile rank of 16%. The comparatively inferior performance of ShortTerm-MinProb-Portia could potentially be attributed to its limited sample size, comprised of 9 samples compared to the 11 samples utilized for long-term models. As larger sample sizes invariably offer a more robust dataset for model learning, this discrepancy could account for the difference in performance. Despite ShortTerm-MinProb-Portia failing to demonstrate statistical significance, we still retained this model for further examination throughout the study. However, we intend to proceed with caution while interpreting the results generated by the short-term response model.

### 3.3 DE proteins as the regulators of GRN

In this study, we built our GRN models utilizing a subset of proteomics data specific to DE proteins. This approach operates under the implicit assumption that DE proteins predominantly govern the regulatory system. Moreover, we relied on nominal p-values to discern DE proteins, as adjusted p-values did not yield significant results (see section ‎3.1). This exclusive dependence on nominal p-values could potentially result in misidentification of DE proteins. In addition, the data contained only one measurement at each time point, which can reduce the performance of DE analysis. To address the uncertainties inherent in selecting DE proteins as system regulators, we compared our chosen models with random models constructed from arbitrarily selected regulatory proteins. Accordingly, we randomly selected 100 sets of 50 proteins (analogous to the count of actual DE proteins) as regulators and reconstructed the network using the chosen models of LongTerm-MinProb-Portia and ShortTerm-KNN-Portia. Our findings indicated that the model based on DE proteins for the long-term response significantly outperformed the random models by a 4% margin, as depicted in Fig. 3–E, suggesting that DE proteins are not only highly responsive to Mg^2+^ ion treatment but also play a pivotal role in the regulatory system. This outcome serves to strengthen the validity of our GRN models. Similarly, for the short-term response, the model based on the DE proteins surpassed random models by 14% (see Fig. 3-E). While less significant to the long-term model, this further underscores the superior role that DE proteins play in the regulation of the system compared to randomly selected proteins.

### 3.4 Performance evaluation of the GRN models

Our GRN models only partially retrieved known links (see Fig. 3-C), with retrieval rates of 23% for ShortTerm-MinProb-Portia and 27% for LongTerm-KNN-Portia, respectively. This comparatively low percentage can be attributed to several factors. In particular, the STRING database, being a consolidation of information from diverse sources, may involve different cell lines and culture techniques. As such, the information retrieved may not be fully applicable to our study's experimental setup. In a related context, Yuan et al. [39] constructed a similar data-driven approach, utilizing the CommonPathway database to compute the percentage of known links retrieved. They reported an approximate success rate of 30%. Importantly, their GRN models were built using an extensive dataset from 89 perturbation conditions. The similarity of our results with their outcomes, despite the smaller sample size in our study, further underscores the credibility of our GRN model.

### 3.5 Mg^2+^ ions alters protein regulatory roles in the network

**Fig. 4:**
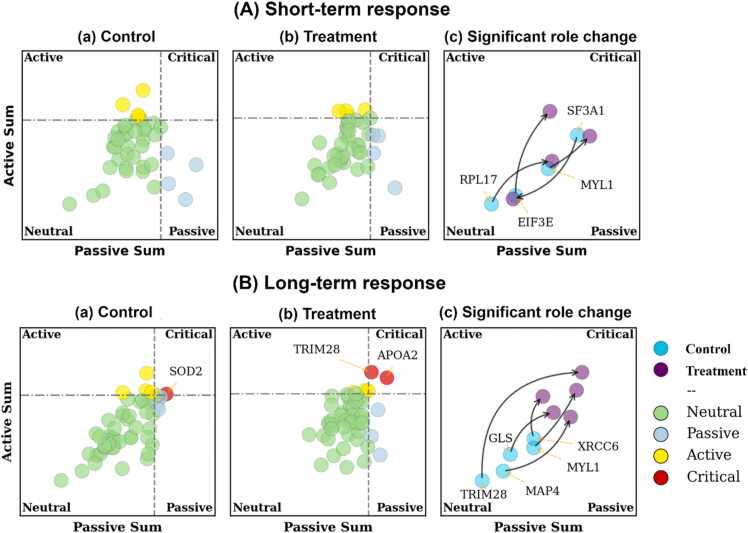
The results of the protein role analysis for the selected model of (A) the short-term and (B) the long-term response. The dashed lines in the plots indicate the upper 75th percentile on each axes. Column (3) shows the significant protein role change across control and Mg^2+^ ions treatment. Gene names are used instead of protein names.

We investigated the impact of Mg^2+^ ions on the protein regulatory roles in the network using PRA, as depicted in Fig. 4. In the case of short-term response, the VSA did not identify any critical proteins for either group (refer to Fig. 4-A-a and A-b). Proteins with significant role changes between the two groups are displayed in Fig. 4-A-c, which include SF3A1, RPL17, MYL1, and EIF3E. For the long-term response, the VSA pinpointed SOD2 as the critical protein in the control group (refer to Fig. 4-B-a), while TRIM28 and APOA2 emerged as critical proteins in the treatment group (Fig. 4-B-b). Proteins exhibiting significant role changes between the two groups are presented in Fig. 4-B-c, comprising MYL1, XRCC6, MAP4, TRIM28, and GLS.

### 3.6 Mg^2+^ ions impact protein-protein regulatory relationships

**Fig. 5:**
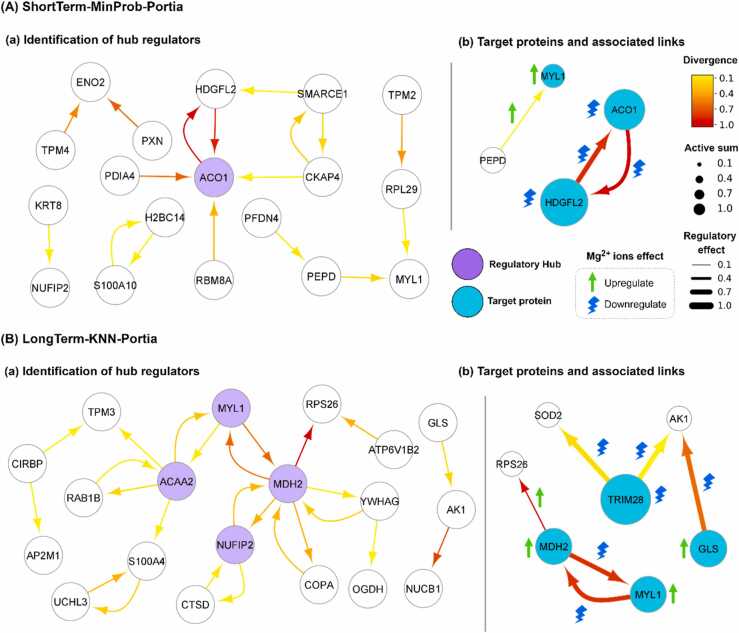
The significance of divergence in the regulatory links due to Mg^2+^ ion treatment is given in the first column, A-a and B-a. The color of the edges shows the significance of the change from control to Mg^2+^ ions treatment. The active sum shows the regulatory influence of a protein on the rest of the network. The regulatory effect shows the strength of the regulation. These two complementary indications are only for the second column (A-b and B-b). Gene names are used instead of protein names. The graph was created using Cytoscape [51].

We investigated the impact of Mg^2+^ ions on the protein regulatory relationships using PRDA. The first column in Fig. 5 depicts the top 10% of the most significantly altered interactions by the treatment. Our analysis emphasized proteins with a central role in the network, as indicated by their involvement in multiple significant interactions. Proteins engaged in four or more significant interactions were classified as regulatory hubs within the network. In the short-term response (see Fig. 5-A-a), the analysis identified ACO1 as the sole regulatory hub within the network. However, ENO2 was engaged in the two most significantly impacted connections in the network, indicating its potential importance. Similarly, while HDGFL2 was not classified as a regulatory hub, it exhibited significant interactions with ACO1, suggesting its important involvement in the response mechanism. In the context of the long-term response (see Fig. 5-B-a), we identified MDH2, MYL1, NUFIP2, and ACAA2 as the regulatory hubs, which predominantly influenced the network through their substantial interactions with each other and the rest of the network. Notably, the mutual interactions of MDH2 with RPS28 and MYL1 ranked among the most significantly altered interactions due to the treatment.

### 3.7 Principal protein targets of Mg^2+^ ions

The combined results of PRDA and the PRA suggest the seven proteins of SF3A1, RPL17, MYL1, EIF3E, ACO1, HDGFL2, and ENO2 as the main effectors of the Mg^2+^ treatment on the short-term response (see Fig. 5-A-a and Fig. 4-A-c). Amongst them, we identify ACO1, HDGFL2, and MYL1 as the primary molecular targets of Mg^2+^ ion treatment, rationalized as follows. ACO1 and HDGFL2**,** as they were the most dominant proteins in the regulatory network identified by the PRDA (see Fig. 5-A-a) and were also among the top 25% largest role change determined by VSA (results were not shown). MYL1**,** as was identified as a significant protein identified by VSA (see Fig. 4-A-c) and was part of the shortlisted network identified by the PRDA (see Fig. 5-A-a).

In the long-term response, the seven proteins of MYL1, XRCC6, MAP4, TRIM28, GLS, MDH2, NUFIP2, and ACAA2 were identified as the primary effectors of Mg^2+^ ions (see Fig. 5-B-b and Fig. 4-B-c). Among these, we particularly highlight MDH2, TRIM28, MYL1, and GLS as the principal molecular targets of Mg^2+^ ion treatment in the long-term response, rationalized as follows. MDH2, as it emerged as the most dominant protein in the regulatory network as per PRDA results (see Fig. 5-B-a). TRIM28, as it was identified as the most significant protein by VSA, exhibiting the largest role change from control to treatment conditions (see Fig. 4-B-c). Moreover, this protein showed a role change from neutral to critical upon Mg^2+^ ions treatment (see Fig. 4-B), indicating its potential significance in response to the treatment. MYL1, on account of its identification as a significant protein in both the PRDA and VSA (see Fig. 5-B-a and Fig. 4-B-c). GLS, as it was recognized as a significant protein using the VSA (see Fig. 4-B-c) and was also part of the shortlisted network identified by the PRDA (see Fig. 5-B-a).

### 3.8 Interpretation of the target proteins and comparative analysis with previous reports

We provide background information regarding MDH2, TRIM28, MYL1, and GLS as the principal molecular targets of Mg^2+^ ion treatment in the long-term response. These proteins are identified in the UniProt database [52] by their respective accession numbers: MDH2 (P40926), TRIM28 (Q13263), MYL1 (P05976), and GLS (O94925). We do not assess the principal proteins of the short-term response, as the corresponding model did not yield statistical significance.

MDH2, an abbreviation for Malate Dehydrogenase, is a mitochondrial oxidoreductase involved in the conversion of malate to oxaloacetate [53]. This reversible process uses the NAD-NADH cofactor system, making MDH2 a key player in the tricarboxylic acid cycle (TCA), also known as the Krebs Cycle, a fundamental aspect of cellular energy metabolism [53]. The refolding efficiency of MDH2 has been observed to increase in the presence of ATP [54]. Additionally, isoforms of the enzyme NAD Malate Dehydrogenase in plant species exhibit dependency on magnesium concentration, impacting energy and carbohydrate metabolism [55], [56]. Moreover, in cyanobacterial TCA, MDH activity can be augmented by magnesium, suggesting a potential regulatory role of magnesium in energy and carbohydrate metabolism with MDH as a target [57].

TRIM28, the gene encoding the protein transcription intermediary factor 1 (TIF1)-beta, plays a multifaceted role in cellular functions pertaining to gene expression and translation [52]. It achieves this through its ability to bind to DNA and RNA, and its capacity to modify histones. TRIM28 is implicated in binding to and potentially regulating tumor promoters associated with the initiation and progression of osteosarcoma. Targeting this signaling pathway holds promise for osteosarcoma treatment [58]. TRIM28 also contributes to skeletal development by stabilizing skeletal stem cells, indicating its involvement in maintaining skeletal integrity and function [56].

TIF1 proteins act as co-repressors, binding to vertebrate-specific Krüppel-associated box (KAP) factors, which are known for their potent transcriptional repressor activity [59]. Specifically, KAP1 has been shown to induce the RUNX family transcription factor 2 (Runx2), facilitating the activation of osteoblast phenotype transition [60]. Consequently, KAP1 may serve as a secondary target for magnesium-related cell differentiation within osteogenic lineages [60]. TIF1 binding to heterochromatin protein and the subsequent deacetylation of histones leads to gene silencing, further emphasizing its critical role in gene expression regulation [61]. TIF1 has been shown to be crucial for maintaining the stability of hematopoietic stem cells [62], yet its comparable function in mesenchymal stem cells remains unclear.

MYL1, standing for Myosin Light Chain 1, is a protein that, while not having regulatory properties, is crucial for the formation and maintenance of myosin fibers [52]. It plays a vital role in skeletal muscle formation and is critical for skeletal muscle function [63], [64]. MYL1 is part of the Myosin group, which have the ability to bind to and hydrolyze ATP [65]. Notably, in a gene expression analysis utilizing clinical data, MYL1 emerged as a potential target for predicting the prognosis of osteosarcoma [66].

GLS, the gene encoding the protein Glutaminase kidney isoform, mitochondrial, is responsible for catalyzing the initial step in the metabolism of the amino acid, Glutamine [52]. This enzyme plays a significant role in transforming glutamine into glutamate, which is associated with metabolic pathways [52], [67]. In particular, GLS has been linked to the tricarboxylic acid cycle, which is a central part of cellular metabolism [67]. This enzyme's function has also been linked to cancerous processes. For instance, its catalysis leads to an increase in alpha-ketoglutarate, a crucial nutrient for leukemia cells, thereby implicating it in the proliferation of this type of cancer [68]. Studies have shown a relationship between solubilized form of GLS from rat liver mitochondria and magnesium concentrations, where an increase in magnesium concentration stimulated the enzyme activity [69]. Similar results were reported using in vitro ascites cell line [70]. Furthermore, bacterial Glutaminase also displays magnesium-dependent activity [71]. Consequently, GLS could potentially serve as a target for magnesium stimulation, although the repercussions for mesenchymal stem cell proliferation and osteogenesis remain unknown.

In the provided background, three proteins of MDH2, MYL1, and GLS have been directly associated with ATP and metabolic functions. Given that ATP synthesis requires Mg^2+^ ions [72], a general association between these proteins and Mg^2+^ ions is expected. In addition, MDH2, GLS, and TRIM28 were shown to be differentially expressed in MSCs in response to Mg^2+^ ions in the studies of Sánchez et al. [25] and Omidi et al. [26]. These studies had similar methodology; they utilized HUCPV cells for the culture and employed mass spectrometry for proteomic extraction. In particular, Sánchez et al. used Mg^2 +^ extracts with a concentration of 6.08 mM, similar to the 5 mM used in our study. This agreement in the results further signifies the importance of these proteins and the validity of our findings.

In a previous study by Moreno et al. [16], S100A10 and PABPC4 were identified as the target proteins in the short-term (days 1–11) response of MSCs to Mg^2+^ ions through GRN inference and protein role analysis. In our study, these proteins were amongst the DE proteins and identified as important elements in the regulatory network (see Fig. 5-A-a). However, the results of our study showed more important roles for the three proteins of ACO1, HDGFL2, and MYL1 in responding to Mg^2+^ ions. These findings potentially stem from methodological advancements in our approach over that of Moreno et al. More notably, we utilized different GRN inference models, including linear and non-linear regression models as well as precision matrix approach, and chose the best-performing model through model selection, while Moreno et al. solely relied on a linear model to extract the regulatory relationships. Precision matrices, notable for their ability to correct for redundant linear correlations, have been demonstrated to more accurately model regulatory relationships under normality assumption [24]. In addition, Portia refines the precision matrices by fitting one linear regression per target gene, and reweight each putative regulator based on its centrality in the network. This reduces the symmetry of precision matrices as well as produces directed GRNs [24].

### 3.9 Principal protein interactions affected by Mg^2+^ ions

In order to better understand how Mg^2+^ ions influence the identified target proteins, we conducted a detailed analysis of the protein-protein interactions related to these targets. Following a method akin to that used with PRDA, we quantified significant protein interactions resulting from the treatment but concentrated solely on the connections involving the target proteins. The second column in Fig. 5 shows the top 10% of the protein connections associated with target genes, most significantly affected by the treatment. This representation includes the results presented in Fig. 5, but also extends to cover those target proteins that were not detected by the PRDA in section ‎3.6. In the short-term response (see Fig. 5-A-b), the results indicate that the Mg^2+^ treatment weakened the robust interaction between ACO1 and HDGFL2 and augmented the interaction between MYL1 and PEPD. In the long-term response (see Fig. 5-B-b), the treatment diminished the interaction between MDH2 and RPS26, while enhancing its mutual interactions with MYL1. The treatment also strengthened the interactions between GLS and AK1, as well as between TRIM28 and both AK1 and SOD2.

### 3.10 Limitations

In addition to the previously mentioned limitations, namely the sample size and the imputation of the missing values, this study has several other important limitations. First, the proteomics data used in this study was limited to only one measurement per time point. Although time-series analysis enabled us to determine DE proteins, having multiple replicas per measurement could improve the robustness of our statistical analysis. Secondly, our study limited the evaluation of Mg^2+^ ions to a concentration of 5 mM. However, the in vivo implant degradation process could potentially yield higher concentrations, as high as 40 mM [8]. Therefore, to fully comprehend the dose-dependent effects of Mg^2+^ ions, additional investigations incorporating a broader spectrum of Mg^2+^ ion concentrations should be conducted. Third, we utilized Portia for our time series data, although it was originally designed for stationary data [24]. We made the simplifying assumption that protein decay occurred equally across different measurement days. Despite this simplification, Portia outperformed other methods and produced robust results (see Fig. 3-D). Fourth, we did not evaluate the performance of GRN inference using other common techniques such as the receiver operating characteristic curve, which accounts for both true positive and false positive rates. This was because, in our study, defining false positives correctly is impractical since not all protein links are characterized in our target cell, human MSCs. Fifth, we assumed that DE proteins, as transcription factors, directly encoded protein expressions, which is a common assumption in the literature [20], [24], [43]. However, protein expression involves two stages: transcription and translation. It is plausible that for certain expression profiles, the overlooked stage of gene translation played a significant role in the overall expression process.

## 4 Conclusion

In this study, we employed GRN analysis to investigate the regulatory effect of Mg^2+^ ions on the molecular response of MSCs. MSCs play a pivotal role in bone regeneration by proliferating and differentiating into various cell types essential for repair. Our results provide insights into the key proteins and protein-protein interactions that are involved in the cellular response of MSCs to Mg^2+^ ions, shedding light on the regulatory role of Mg^2+^ ions on MSCs. The results of our study identified the four proteins of MYL1, MDH2, GLS, and TRIM28 as the primary targets of Mg^2+^ ion treatment in long-term response (days 1–21) (see Fig. 5-B-a). Furthermore, we discovered critical protein interactions influenced by Mg^2+^ ions, including MDH2-MYL1, MDH2-RPS26, TRIM28-AK1, TRIM28-SOD2, and GLS-AK1. Considering the established role of Mg^2+^ ions in regulating MSC proliferation and osteoblastic differentiation, we speculate that these proteins may be crucial players in these processes. However, further verification is necessary to confirm their specific roles. Once verified, these results could assist in the optimization of Mg-based biomaterials in promoting bone regeneration and improving implant integration.

## Statement

During the preparation of this work the author(s) used ChatGPT to revise the text and grammar checking. After using this tool/service, the author(s) reviewed and edited the content as needed and take(s) full responsibility for the content of the publication.

## CRediT authorship contribution statement

**Hartmuth Schlüter:** Funding acquisition. **Christian J. Cyron:** Funding acquisition, Supervision, Writing – review & editing. **Roland C. Aydin:** Formal analysis, Writing – review & editing. **regine willumeit romer:** Funding acquisition. **Berit Zeller-Plumhoff:** Conceptualization, Supervision, Writing – review & editing. **Jalil Nourisa:** Conceptualization, Data curation, Formal analysis, Investigation, Methodology, Software, Validation, Visualization, Writing – original draft. **Heike Helmholz:** Formal analysis, Validation, Writing – original draft. **Antoine Passemiers:** Formal analysis, Software, Validation, Visualization, Writing – review & editing. **Farhad Shakeri:** Formal analysis, Software, Visualization. **Maryam Omidi:** Methodology. **Daniele Raimondi:** Writing – review & editing. **Yves Moreau:** Funding acquisition. **Sven Tomforde:** Supervision.

## Declaration of Competing Interest

The authors declare that they have no known competing financial interests or personal relationships that could have appeared to influence the work reported in this paper.
